# Group-Based Suicide Safety Planning and Skills Training for Veterans With High Suicide Risk: A Randomized Clinical Trial

**DOI:** 10.1001/jamanetworkopen.2026.15029

**Published:** 2026-05-29

**Authors:** Marianne Goodman, Shari Jager-Hyman, Maureen Monahan, Sofie Glatt, Michael E. Thase, Shiela O’Brien, Alison Krauss, Hanga C. Galfalvy, Sarah R. Sullivan, James Luther, Gregory K. Brown

**Affiliations:** 1 Veterans Integrated Service Network (VISN) 2 Mental Illness Research, Education, and Clinical Center (MIRECC), James J. Peters Veterans Affairs Medical Center, Bronx, New York; 2 Department of Psychiatry, Icahn School of Medicine at Mount Sinai, New York, New York; 3 Department of Psychiatry, Perelman School of Medicine, University of Pennsylvania, Philadelphia; 4 New York State Psychiatric Institute, New York; 5 Department of Psychiatry, Vagelos College of Physicians and Surgeons, Columbia University, New York, New York; 6 Corporal Michael J. Crescenz Veterans Affairs Medical Center, Philadelphia, Pennsylvania; 7 Central Texas Veterans Health Care System, Waco; 8 Department of Psychology, Hunter College and the City University of New York Graduate Center, City University of New York, New York; 9 Department of Epidemiology, University of Pittsburgh, Pittsburgh, Pennsylvania; 10 VISN 5 MIRECC, Pittsburgh Veterans Affairs Medical Center, Pittsburgh, Pennsylvania

## Abstract

**Importance:**

Novel evidence-based suicide-specific treatments are critical to addressing rising rates of suicide among veterans.

**Objective:**

To determine whether Project Life Force (PLF), a group intervention that augments suicide safety planning (SSP) with skills training, plus treatment as usual (TAU) delays or decreases suicidal behavior compared with TAU alone.

**Design, Setting, and Participants:**

This multisite randomized clinical trial, using intention-to-treat analyses, included veterans at high risk for suicide. Participants were recruited after psychiatric inpatient discharge or referral from 4 Veterans Health Administration outpatient care teams in hospitals in Pennsylvania, New York, and Texas between March 2018 and February 2024. Data were analyzed from April 2024 to October 2025.

**Intervention:**

PLF is a manualized, 10- to 12-session (75-90 minutes per session) group intervention combining SSP with distress tolerance, emotion regulation, and interpersonal skills training. Sessions cover each of the 6 SSP steps, health management, reasons for living, SSP accessibility, and a recap, with up to 4 optional booster sessions offered. All participants received TAU, constituting individual SSP and standard outpatient mental health care.

**Main Outcomes and Measures:**

The primary outcome was time in days to first suicidal behavior (aborted, interrupted, or actual attempts or suicide death) over 12 months, assessed using the Columbia Suicide Severity Rating Scale and chart review. Secondary outcomes over 12 months included self-report measures of hopelessness, depression, treatment use, attitudes toward help-seeking, and suicide-related coping.

**Results:**

A total of 207 participants (mean [SD] age, 46.1 [13.9] years; 178 male [86%]) were assessed at baseline and randomly assigned to PLF plus TAU (101 participants) or TAU alone (106 participants). Cox proportional hazards models showed no significant between-group difference in time to suicidal behavior; however, compared with TAU, PLF participants had a lower hazard of actual suicide attempts over 1 year (HR, 0.49; 95% CI, 0.26-0.92; *P* = .03). Secondary outcome analyses included data from 132 participants (64%) at posttreatment, 125 (60%) at month 6, and 130 (62%) at month 12. Treatment-by-time effects ranged from Cohen *d* = −0.03 to 0.35. Notably, PLF significantly improved suicide-related coping at posttreatment (estimate, 4.06; Cohen *d*, 0.21; *P* = .04) and month 6 (estimate, 5.36; Cohen *d*, 0.27; *P* = .007). No study-related adverse events occurred.

**Conclusions and Relevance:**

In this randomized clinical trial of 207 participants, PLF plus TAU did not change time to suicidal behavior but did increase time to actual suicide attempts and improve suicide-related coping, thereby enhancing SSP impact.

**Trial Registration:**

ClinicalTrials.gov Identifier: NCT03653637

## Key Points

**Question:**

Does Project Life Force (PLF), a manualized suicide safety planning group intervention augmented with skills training, reduce suicidal behavior among veterans at high risk for suicide over 12 months?

**Findings:**

In this randomized clinical trial of 207 veterans at high risk for suicide, no difference was found in time to first suicidal behavior between participants in the PLF arm compared with those in the treatment as usual (TAU) arm. However, PLF participants demonstrated a significantly lower number of actual attempts and improved suicide-related coping than TAU participants.

**Meaning:**

These findings suggest that PLF may lower suicide risk among veterans at high risk for suicide.

## Teaser

This randomized clinical trial evaluates Project Life Force, a group-based suicide safety planning and skills training intervention for veterans at high risk for suicide, comparing its effects to standard treatment over a 12-month period.

## Introduction

Compared with civilians, US veterans have more than twice the age- and sex- adjusted suicide rate and account for about 15% of suicide deaths.^1^ Despite suicide prevention being a top clinical priority for the US Department of Veterans Affairs (VA), more than 6400 veterans died by suicide in 2022.^1^ One component of VA’s suicide prevention efforts mandates that clinicians develop with every patient at high risk a Suicide Safety Plan (SSP) to mitigate short-term suicide risk.^2,3^ Safety planning is strongly recommended by several government agencies^4,5^ and associated with improvements in suicidal symptoms, depression, hopelessness, treatment engagement and hospitalization rates.^6^

However, significant obstacles limit the usefulness of SSPs,^7^ including limited clinician follow-up after SSP development^8^ and inadequate skills and/or support networks necessary to optimize SSP implementation.^9^ To address these gaps, we created Project Life Force (PLF), a novel group intervention that leverages social interaction and skills training to maximize SSP engagement and effectiveness.^10,11^

PLF is a manualized 10-session group intervention wherein veterans revise their SSPs while learning distress tolerance, emotion regulation, and interpersonal skills necessary for effective implementation (trial protocol in Supplement 1).^10,11^ PLF uses a group format to mitigate loneliness and foster belongingness, both key risk factors for suicide.^12,13^ Advantages of group therapy for veterans at high risk for suicide include cost-effectiveness; use of peer support, which has an emerging evidence base for suicide prevention^14^; and congruency with military cultural values of teamwork and camaraderie.^15^ PLF differs from many skill-based group treatments as suicidality is directly discussed to emphasize the application of coping skills and management. Discussion of shared experiences and reciprocal support cultivates connection, belongingness, and self-efficacy.

After our open-label PLF study that demonstrated improvements in suicidal symptom severity, depression, and hopelessness,^11^ we conducted the current multisite RCT and now report on our first 2 aims described in our protocol paper^10^ and clinical trial registration. Our objective was to compare the impact of PLF plus TAU (henceforth referred to as PLF) with TAU on suicide behavior and associated suicide risk factors, including hopelessness and depression, as well as protective factors including mental health care use and suicide-related coping (SRC). We also explored a potential mediator of PLF, thwarted belongingness, based on the interpersonal theory of suicide.^12,13^ We hypothesized that, relative to TAU, PLF would (1) increase the time to suicide behavior, (2) decrease depression and hopelessness, (3) increase positive attitudes toward mental health treatment and improve engagement, (4) increase SRC, and (5) decrease thwarted belongingness.

## Methods

### Study Design

This RCT was conducted at 4 VA Medical Centers (VAMCs): James J. Peters VAMC (Bronx, New York; n = 129), Corporal Michael J. Crescenz VAMC (Philadelphia, Pennsylvania; n = 59), Central Texas Veterans Health Care System (Waco, Texas; n = 17), and Northport VAMC (Northport, New York; n = 2). Study procedures were reviewed and approved by each VAMC’s Institutional Review Board and Research and Development Office. The study was registered at ClinicalTrials.gov and adheres to the Consolidated Standards of Reporting Trials (CONSORT) reporting guidelines.

### Participants and Procedures

**Figure 1.**
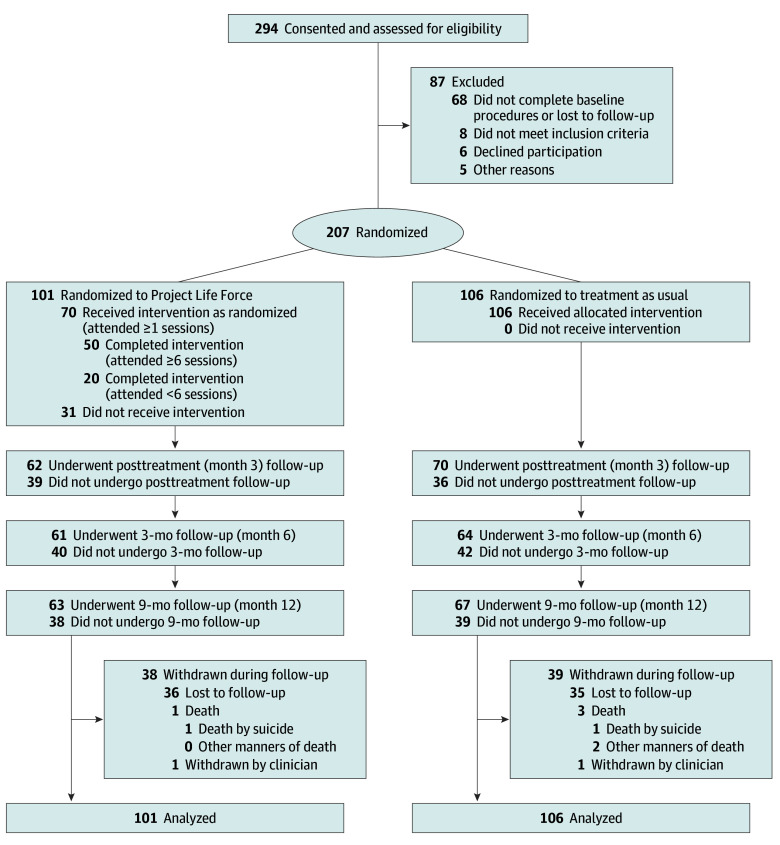
CONSORT Flow Diagram

A total of 294 veterans at high risk for suicide provided written informed consent after a suicide-related hospitalization or referral from an outpatient clinic. Among these individuals, 207 were randomized to either PLF or TAU using a computerized block randomization stratified by suicide attempt history (yes or no). All randomized participants were included in analyses consistent with the intention-to-treat (ITT) principle. Inclusion and exclusion criteria are detailed elsewhere.^10^ Assessments were collected at baseline, posttreatment (month 3), 3 months posttreatment (month 6), and 6 months posttreatment (month 12). Figure 1 presents a CONSORT flow diagram.

### Treatment Conditions

#### TAU

TAU was operationalized as current standard of care for individuals with suicidal thoughts or behavior who were discharged from an inpatient unit or flagged for high risk for suicide, including individualized safety planning. The only methodological difference between conditions is that PLF participants also attended the PLF group sessions to augment their SSPs.

#### PLF

PLF is a manualized 10-week group treatment with weekly 90-minute sessions. Session content is described in Goodman et al.^10^ In this RCT, PLF was cofacilitated by 2 licensed mental health clinicians and included 4 to 6 veterans. After completing the PLF curriculum, veterans were offered optional booster sessions (n = 4).

#### Fidelity Monitoring

Independent PLF experts assessed fidelity by rating a random selection (20%) of session recordings using the PLF Adherence and Competence Monitoring Rating Form, developed for the study. The form evaluates general clinical competence and session-specific PLF components on a scale of 0 to 3 points. Adherence was 94% for general competency and 90% for session-specific adherence averaged across sites.

### Protocol Modifications

This RCT, conducted from March 2018 to February 2024, was affected by the COVID-19 pandemic, necessitating protocol modifications affecting participants (n = 119; 58 TAU and 61 PLF). After March 2020, PLF moved from in-person to virtual delivery, although session content did not change. Modifications necessary for virtual PLF included having a communication coordinator who supported virtual logistics and developing virtual safety protocols. Additionally, a prespecified exploratory aim assessing SSP quality was not completed due to difficulties obtaining revised SSPs with the transition to virtual care.

### Measures

#### Primary Outcome: Time to Suicide Behavior

Suicide behavior was operationalized as a composite outcome defined as any occurrence of suicide behavior (actual, interrupted, or aborted suicide attempt or suicide death) ascertained from 3 sources (self-report, record review, and death registry) during the 12-month follow-up period. First, the Columbia Suicide Severity Rating Scale,^16^ the gold standard for suicide assessment,^17^ was administered at every study visit. Using the behavior subscale, we defined presence of suicide behavior, in part, as an affirmative response for any actual, aborted, or interrupted attempt. We randomly selected 20% of C-SSRS recordings to assess interrater reliability, with adequate intraclass correlations ranging from 0.87 to 0.93 across sites. Second, we extracted instances of suicide behavior from patient charts for all participants who missed study assessments. Third, to measure suicide behavior that included suicide deaths for participants who were lost to follow-up, in addition to examining VA medical records, we performed internet searches, queried state vital statistics registries, and reviewed death certificates as needed.

#### Secondary and Exploratory Outcomes

Depression was assessed using the Beck Depression Inventory-II.^18^ Hopelessness was measured using the Beck Hopelessness Scale,^19^ with additional separate sum scores for negative and positive expectations.^20^ Mental health treatment engagement was determined using chart reviews to quantify the sum of outpatient mental health treatment visits for 6 months, excluding PLF sessions. During the pandemic, this included telephone and virtual sessions. Attitudes toward seeking professional help for psychological disturbance was measured by the Attitudes Toward Seeking Professional Psychological Help Scale–Short Form.^21,22^ We used the Suicide-Related Coping Scale (SRCS)^23^ to measure knowledge of, and perceived confidence in using, internal coping strategies and external supports to regulate suicidal thoughts and urges. We calculated a total SRCS score, as well as external and internal coping subscales. The exploratory outcome of thwarted belongingness was assessed with the Interpersonal Needs Questionnaire (INQ)-15.^13^

#### Diagnostic and Descriptive Measures

We assessed lifetime and current psychiatric disorders using The Mini-International Neuropsychiatric Interview.^24^ Monitoring and oversight of the diagnostic assessment was performed by investigators from Columbia University.

Past-year alcohol use was assessed using the Alcohol Use Disorders Identification Test-Concise (AUDIT-C),^25^ a brief 3-item alcohol screening tool. Past-year substance use was measured using Drug Abuse Screening Test (DAST-10),^26^ a self-report measure with 10 binary items (0 or 1). Participant race and ethnicity was self-reported; this demographic information is collected routinely in our studies because treatment response and suicide risk has differed across racial and ethnic groups. We did not collect additional information about the specific racial groups in the other race category.

### Statistical Analysis

#### Primary Outcome

Data were analyzed from April 2024 to October 2025. For our primary outcome of composite suicide behavior, we used a Cox proportional hazards survival regression model^27,28^ to calculate hazard ratios (HRs). All primary analyses were conducted under the ITT framework. Analyses were adjusted for enrollment site and lifetime suicide attempt history at baseline. Treatment condition was the primary variable. Time to event was defined as the number of days from randomization to the first suicide behavior. Because the first occurrence of suicide behavior per participant was included in the analysis, each event reflects a unique participant. Participants with no suicide behavior during the follow-up period were right censored at 365 days, and those with incomplete follow-up (and without data in their medical records) were right censored at their last available day. Kaplan-Meier estimates of the cumulative hazard function for both groups were plotted, and the proportional hazards assumption was verified with the Grambsch and Therneau^29^ test for the global model. Analyses were conducted using R, version 4.4.2 (R Core Team), and 2-sided *P* values less than 0.05 were considered statistically significant.

#### Post Hoc Analysis With Actual Suicide Attempts

We also used the previously described analytic strategy with actual suicide attempt as the sole outcome.^16^ This analysis was not prespecified and should be considered exploratory.

#### Secondary and Exploratory Outcomes

Secondary (ie, depression severity, hopelessness, attitudes toward seeking professional psychological help, outpatient mental health care use, and SRC) and exploratory (ie, thwarted belongingness) outcomes were analyzed using linear mixed effects (LME) regression models. All LMEs included fixed effects for randomization condition, time (treated as a factor, with baseline as the reference point), their interaction, and an adjustment for site (with the primary site as the reference). The treatment effect of PLF vs TAU was operationalized as the treatment-by-time interaction. Treating study time point (months 3, 6, and 12) as a factor allowed direct comparisons of treatment effects at each individual time point rather than assuming an overall linear trend. LMEs accommodate dropout or missing data under the assumption of missing at random, which was verified using Little’s ^30^ missing completely at random tests (*P* > .05 for all time points). All models were fit with the ITT principle. After these outcome models, we examined the direct effect of treatment condition on belongingness using the mixed-effects models described above. Mediation analyses were conducted only if this effect was statistically significant. In secondary analyses, models with primary, secondary, and exploratory outcomes were conducted with a modified ITT sample consisting of those that attended at least 1 session of PLF with TAU.

## Results

The sample included 207 participants (101 randomized to PLF and 106 to TAU), among whom 178 (86.0%) were male and 29 (14.0%) were female, and the mean (SD) age was 46.1 (13.9) years. One participant (0.5%) self-identified as American Indian or Alaska Native, 4 (1.9%) as Asian, 71 (34.3%) as Black or African American, 69 (33.3%) as Hispanic or Latino, 80 (38.6%) as White or Caucasian, 26 (12.6%) as multiracial, and 19 (9.2%) as other race. Participant characteristics and descriptives are presented in Table 1. No study-related adverse events occurred.

**Table 1.** Baseline Demographic and Clinical Characteristics (N = 207)^a^

Characteristic	No. (%)
Sex	
Men	178 (86.0)
Women	29 (14.0)
Race	
American Indian or Alaska Native	1 (0.5)
Asian	4 (1.9)
Black or African American	71 (34.3)
White or Caucasian	80 (38.6)
Multiracial	26 (12.6)
Other^b^	19 (9.2)
Ethnicity	
Hispanic or Latinx	69 (33.3)
Non-Hispanic or non-Latinx	124 (59.9)
Education level	
Some high school	1 (1.4)
High school diploma or GED	54 (26.1)
Some college or 2-y degree	104 (50.2)
Bachelor’s degree	30 (14.5)
Master’s degree	5 (2.4)
Employment status	
Full-time	36 (17.4)
Part-time	8 (3.9)
Unemployed	79 (38.2)
Student	12 (5.8)
Retired	54 (26.1)
Self-employed	5 (2.4)
Marital status	
Married or cohabiting with someone as if married	46 (22.2)
Widowed	4 (1.9)
Divorced or annulled	57 (27.5)
Separated	33 (15.9)
Never married	57 (27.5)
Baseline treatment status	
Not currently receiving psychiatric treatment	4 (1.9)
Receiving outpatient mental health care	93 (44.9)
Current psychiatric hospitalization	90 (43.5)
Partial hospitalization program or other institutional living situation	9 (4.4)
Family member or close friend supports treatment	
Yes	158 (76.3)
No	37 (17.9)
No. of offspring	
0	66 (31.9)
1-2	74 (35.7)
≥3	86 (42.5)
Lives alone	
Yes	108 (52.2)
No	88 (42.5)
Suicidal ideation or suicide attempt before military	
Yes	41 (19.8)
No	154 (74.4)
Served time in prison	
Yes	41 (19.8)
No	147 (71.0)
Psychiatric diagnosis at baseline^c^	
Current major depressive episode	
Yes	156 (75.4)
No	26 (12.6)
Past major depressive episode	
Yes	117 (56.5)
No	65 (43.5)
Posttraumatic stress disorder	
Yes	136 (65.7)
No	61 (29.5)
Generalized anxiety disorder	
Yes	124 (59.9)
No	67 (32.4)
Antisocial personality disorder	
Yes	42 (20.3)
No	155 (74.9)
Past substance abuse	
Yes	71 (34.3)
No	116 (56.0)
Past alcohol abuse	
Yes	62 (30.0)
No	124 (59.9)
Lifetime suicidal behavior or attempt history at baseline^d^	
Aborted suicidal behavior	
Yes	59 (28.5)
No	136 (65.7)
Interrupted suicidal behavior	
Yes	42 (20.3)
No	154 (74.4)
Actual suicide attempt^e^	
Yes	151 (73.0)
No	55 (26.6)

Abbreviations: C-SSRS, Columbia-Suicide Severity Rating Scale; GED, general education degree.

^a^ Responses were optional, so category responses and percentages per category do not total 207 or 100%.

^b^ This category includes any race that was not designated above. The study did not collect additional information about the specific racial groups in this category.

^c^ Assessed with the Mini-International Neuropsychiatric Interview.^24^

^d^ Assessed with the C-SSRS.^16^

^e^ Because lifetime actual attempts were a covariate in the primary analysis, information for individuals missing the C-SSRS was obtained using chart reviews.

### PLF Session Attendance

Among the 101 veterans randomized to PLF, 70 (69.3%) attended at least 1 session, of whom 46 (65.7%) completed at least 6 group sessions. The remaining 31 veterans (30.7%) did not attend any PLF sessions. Table 2 presents session attendance and treatment trial descriptive data.

**Table 2.** Treatment Trial Descriptive Data

Variable	PLF (n = 101)	TAU (n = 106)	Total (N = 207)
First suicidal behavior, No. (%)^a^			
Any suicidal behavior	22 (21.8)	29 (27.4)	51 (24.6)
Actual suicide attempt	15 (14.9)	28 (26.4)	43 (20.8)
Aborted attempt	7 (6.9)	1 (0.9)	8 (3.9)
Interrupted attempt	0	0	0
Deaths during the study period, No. (%)			
Suicide deaths	1 (1.0)	1 (0.9)	2 (1.0)
Nonsuicide deaths	0	2 (1.9)	2 (1.0)
PLF sessions attended, No. (%)			
0 Sessions	31 (30.7)	NA	NA
1-5 Sessions	24 (23.8)	NA	NA
6-10 Sessions	36 (35.6)	NA	NA
11-12 Sessions	10 (9.9)	NA	NA

Abbreviations: PLF, Project Life Force; NA, not applicable; TAU, treatment as usual.

^a^ One participant was removed from the survival analyses with these data due to missing information regarding a lifetime suicide attempt, which was adjusted for in those models. Suicidal behaviors (primary outcome) were recorded as described in the Methods section, and all behaviors were submitted to the US Department of Veterans Affairs institutional review board.

### Primary Outcome

**Figure 2.**
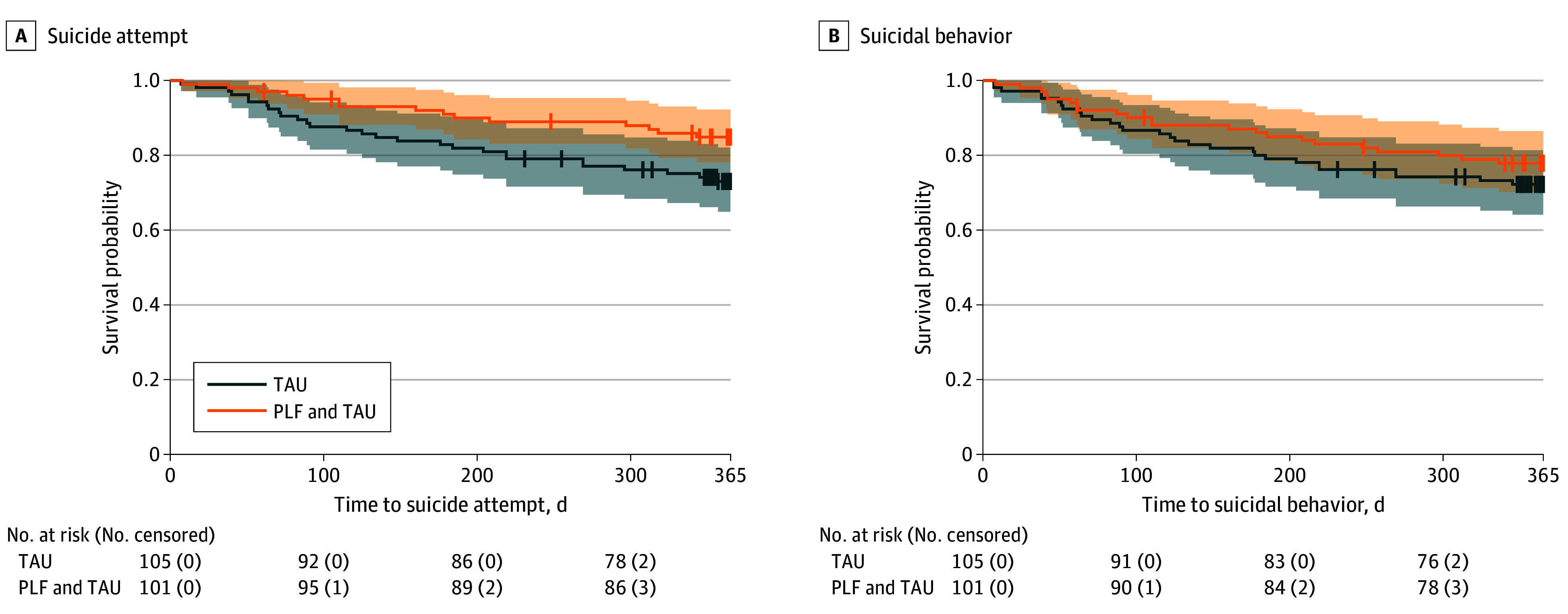
Survival Curves and Risk Tables for Suicidal Behavior and Actual Suicide Attempt Stratified by Treatment Condition Survival probability is plotted over time in days (0-365) to suicidal behavior and suicide attempt for participants randomized to treatment as usual (TAU) and Project Life Force (PLF) plus TAU. Vertical lines denote right-censored data.

During the study period, 51 individuals (24.6%) experienced a suicide behavior composite outcome, including interrupted, aborted, or actual suicide attempt or death by suicide (29 [27.4%] in TAU and 22 [21.8%] in PLF). Among these individuals, 43 (84%) experienced an actual attempt (28 [26.4%] in TAU and 15 [14.9%] in PLF) (Table 2). There were 2 deaths by suicide, 1 in each arm. In the primary survival analysis, treatment condition was not significant for suicide behavior (HR, 0.73; 95% CI, 0.42-1.28; *P* = .27) (Figure 2). Adjustments for site and lifetime suicide attempts were not significant. In the post hoc survival analysis, PLF participants had lower risk than TAU participants of time to first actual attempt (HR, 0.49; 95% CI, 0.26-0.92; *P* = .03) (Figure 2). All model hazards were proportional. The addition of sex as a covariate did not change our findings.

### Secondary and Exploratory Outcomes

Treatment-by-time effects for depression, hopelessness, and outpatient mental health use were nonsignificant (Table 3). Attitudes toward seeking professional psychological help significantly improved at the 3-month follow-up (estimate, 2.14; Cohen *d*, 0.25; *P* = .01), and positive expectations toward the future significantly improved at 9-month follow-up (estimate, 1.17; Cohen *d*, 0.24; *P* = .02) in PLF relative to TAU (Table 3). Overall SRC significantly improved in PLF relative to TAU at posttreatment (estimate, 4.06; Cohen *d*, 0.21; *P* = .04) and 3-month follow-up (estimate, 5.36; Cohen *d*, 0.27; *P* = .007) (Table 3). External coping significantly improved at posttreatment (estimate, 2.09; Cohen *d*, 0.24; *P* = .02) and 3-month follow-up (estimate, 1.74; Cohen *d*, 0.19; *P* = .047), while internal coping significantly improved at 3-month follow-up only (estimate, 2.89; Cohen *d*, 0.35; *P* < .001) for the PLF arm vs the TAU arm. AUDIT-C and DAST-10 scores did not significantly differ at month 12. The INQ subscale of thwarted belongingness was nonsignificant; however, INQ perceived burdensomeness significantly improved at the 3-month follow-up from treatment in PLF relative to TAU (estimate, −3.52; Cohen *d*, −0.20; *P* = .04) (eTable 1 in Supplement 2). Because the direct effect of treatment condition on thwarted belongingness was not significant, exploratory mediation analyses were not pursued further. The addition of sex as a covariate did not change these findings.

**Table 3.** Secondary Outcomes

Variable	BDI-II (n = 207)		BHS (n = 207)		BHS-P (n = 207)		SRCS (n = 207)		SRCS-E (n = 207)		SRCS-I (n = 207)		ATSPPH (n = 205)		Mental health visit (n = 194)	
	Estimate (Cohen *d*)	*P* value	Estimate (Cohen *d*)	*P* value	Estimate (Cohen *d*)	*P* value	Estimate (Cohen *d*)	*P* value	Estimate (Cohen *d*)	*P* value	Estimate (Cohen *d*)	*P* value	Estimate (Cohen *d*)	*P* value	Estimate (Cohen *d*)	*P* value
No. of observations	584	NA	587	NA	587	NA	589	NA	589	NA	589	NA	564	NA	580	NA
Intercept	31.02	<.001^a^	9.21	<.001^a^	5.51	<.001^a^	48.93	<.001^a^	20.12	<.001^a^	21.05	<.001^a^	21.79	<.001^a^	20.89	<.001^a^
Treatment^b^	1.08 (0.06)	.61	0.74 (0.09)	.43	−0.65 (−0.16)	.13	−0.89 (−0.06)	.59	−0.55 (−0.08)	.44	−0.21 (−0.03)	.74	0.45 (0.06)	.57	0.00 (0)	>.99
Month 3	−7.24 (−0.48)	<.001^a^	−1.80 (−0.26)	.009^a^	0.41 (0.12)	.22	1.66 (0.12)	.21	0.69 (0.11)	.24	0.40 (0.07)	.45	−0.39 (−0.07)	.50	−0.68 (−0.04)	.73
Month 6	−5.30 (−0.34)	.001^a^	−1.64 (−0.23)	.02^a^	0.43 (0.12)	.21	0.22 (0.02)	.87	0.60 (0.10)	.32	−0.35 (−0.06)	.53	−1.34 (−0.23)	.03^a^	3.44 (0.18)	.08
Month 12	−8.25 (−0.54)	<.001^a^	−1.72 (−0.24)	.01^a^	0.26 (0.07)	.45	3.36 (0.25)	.01^a^	1.56 (0.26)	.01^a^	1.01 (0.18)	.06	0.08 (0.01)	.89	NA	NA
Site (reference, JJPVAMC)																
CMJCVA	−1.15 (−0.08)	.58	0.33 (0.05)	.72	−0.13 (−0.04)	.75	1.66 (0.15)	.30	0.89 (0.18)	.19	0.59 (0.14)	.31	1.07 (0.19)	.17	−8.83 (−0.50)	<.001^a^
CTVCS	−1.71 (−0.07)	.62	−0.31 (−0.03)	.84	0.36 (0.07)	.61	−0.71 (−0.04)	.79	−0.15 (−0.02)	.90	−0.40 (−0.06)	.68	−1.67 (−0.17)	.22	−6.97 (−0.25)	.09
Treatment × month 3	−0.76 (−0.03)	.73	−0.90 (−0.09)	.37	0.76 (0.16)	.11	4.06 (0.21)	.04^a^	2.09 (0.24)	.02^a^	1.08 (0.13)	.17	1.37 (0.16)	.11	1.92 (0.07)	.50
Treatment × month 6	−2.80 (−0.12)	.21	−0.65 (−0.06)	.53	0.51 (0.10)	.30	5.36 (0.27)	.007^a^	1.74 (0.19)	.047^a^	2.89 (0.35)	<.001^a^	2.14 (0.25)	.01^a^	3.43 (0.12)	.23
Treatment × month 12	−0.73 (−0.03)	.74	−1.36 (−0.13)	.18	1.17 (0.24)	.02^a^	3.44 (0.17)	.08	1.61 (0.18)	.06	1.20 (0.15)	.13	1.18 (0.14)	.17	NA	NA

Abbreviations: ATSPPH, Attitudes Toward Seeking Professional Psychological Help Scale–Short Form; BDI-II, Beck Depression Inventory-II; BHS, Beck Hopelessness Scale; BHS-P, BHS Positive Expectation Subscale; CMJCVA, Corporal Michael J. Crescenz Veterans Affairs Medical Center; CTVCS, Central Texas Veterans Health Care System; E, external; I, internal; JJPVAMC, James J. Peters Veterans Affairs Medical Center; NA, not applicable; SRCS, Suicide-Related Coping Scale.

^a^ Represents a statistically significant value (*P* < .05).

^b^ Treatment is operationalized as PLF plus TAU (coded as 1) vs TAU (coded as 0).

### Secondary Analysis

Modified ITT models for the primary outcome are detailed in eResults 1 and eTable 2 in Supplement 2. Models with the secondary and exploratory outcomes are detailed in eResults 2 and eTable3 in Supplement 2.

## Discussion

This study evaluated whether PLF, a group SSP intervention, reduced time to suicide behavior using a composite of suicide behaviors, including suicide deaths, actual attempts, and aborted and interrupted attempts aggregated from 3 data sources within an ITT framework. Our primary outcome, time to first suicide behavior, did not differ by treatment arm. However, time to first actual suicide attempt was significantly longer for the PLF arm.

Given the relative rarity of actual suicide attempts, the inclusion of aborted and interrupted attempts in a composite outcome served to increase statistical efficiency and trial feasibility.^31^ However, after data collection, an unanticipated discrepancy emerged in the distribution of suicide behavior event types in the composite across the 2 study arms. In TAU, nearly all incidents (28 of 29) were classified as actual suicide attempts. In contrast, PLF included a mix of attempt types (22 total events; 15 actual attempts and 7 aborted).

This divergence suggests that the composite outcome may have obscured clinically meaningful differences between groups. One possible interpretation is that PLF shifted the trajectory of suicide behavior away from more severe outcomes (ie, actual attempts) toward less severe or interrupted behavior. Indeed, individuals with actual attempts have been found to represent higher clinical severity compared with other behaviors.^32^ People who ultimately abort a suicide attempt may follow a different decision-making process than those who do not abort their attempt.^33^

Consistent with this interpretation, the finding for actual suicide attempt emerged from a secondary (post hoc) analysis and should be interpreted cautiously. Our finding suggests a potential mechanism whereby increased coping and safety plan use may interrupt progression to more severe suicide behavior. Thus, our findings reinforce the importance of differentiating among actual, aborted, and interrupted suicide behavior to advance suicide research and prevention considering the meaningful distinctions among these types of behavior.^34^ Distinguishing between these behaviors is a strength of the C-SSRS and, thus, our study methodology.^35,36^

Regarding our secondary outcomes, hopelessness and depression decreased across the trial in both treatment arms, consistent with past suicide prevention RCTs.^37,38^ Other differences favoring PLF were more limited, including improved attitudes about seeking psychological care at month 6 and more positive expectations about the future at month 12. These results may suggest that PLF treatment strategies (ie, homework to share SSP with other clinicians) (eMethods in Supplement 2) strengthen veterans’ connections with their mental health team and foster hope, although these effects were not consistent across time points.

PLF demonstrated significant improvements in overall SRC and self-reported use of external resources (eg, better use of outside supports, reaching out to clinicians, knowledge of emergency resources) at 3 and 6 months, and use of internal coping (items on self-efficacy, internal coping strategies) improved at month 6. These improvements likely followed session content that specifically targeted the teaching of skills relevant to augmenting use of the SSP, and a session centered on the Veterans Crisis Line with homework practice to contact 988 (eMethods in Supplement 2).

Of note, thwarted belongingness was not significant. However, veterans in PLF demonstrated significant decreases in another INQ subscale, perceived burdensomeness. This pattern may reflect the group-based format of PLF and encouragement of open sharing and disclosure about suicidal symptoms each session.^39^ Group therapy facilitates learning from others, supports the sharing of strategies for implementation of each SSP step, and enhances recognition that others are also experiencing suicidal concerns.

Nonadherence was substantial (Table 2). Although this nonadherence may have attenuated treatment effects, this dilution is an inherent feature of ITT analyses, which preserve the benefits of randomization and are considered a best practice for RCTs.^40,41,42,43^ Attrition may be due to logistical constraints with group delivery, preferences for individual-focused treatment, or discomfort with suicide disclosure in a group setting. Future implementation-focused research could study ways to enhance engagement^44^ with strategies such as peer support, given the presumed importance of group cohesion,^45^ and technology enhancements.^46,47^

TAU varied considerably between participants, including variability in individual treatment compliance, treatment, and therapist availability across sites, particularly during the pandemic. In addition, eligible veterans with suicide attempts during the previous year could participate in VA suicide prevention telehealth hubs that launched online near the end of the trial; these specialized psychotherapy treatments greatly enhanced TAU. Although TAU was guided by national VA standards, substantial heterogeneity occurred over time. This variability, particularly during and attributable to the COVID-19 pandemic, may have attenuated between-group differences. Efforts to better characterize and define TAU in future trials may help clarify the unique impacts of add-on treatments.

Lastly, the global pandemic’s impact on the trial cannot be underestimated.^48^ The Bronx site was the epicenter of COVID-19 infections for New York, with all in-person care prohibited except for emergencies and COVID-19 care. The Philadelphia VA site was closed to recruitment and not allowed to conduct research for more than 1 year. Several participants died from COVID-19 complications during the follow-up period. The pandemic also necessitated a transition from in-person to virtual PLF delivery and broadly affected mental health, stress, health care access, and study procedures. These factors were not formally modeled and likely influenced engagement and outcomes.^49,50^

### Limitations

Limitations of the study include a higher-than-expected attrition, particularly between consent and randomization. Participants were often consented during psychiatric hospitalization and scheduled their baseline assessments after discharge; postdischarge is a time of enhanced suicide risk and instability.^51^ Our noncompletion rate was higher than other multisession group interventions,^38,52^ which may be due to pandemic-related factors that diluted the impact of the PLF intervention and also disrupted TAU services. A clearer definition of TAU is warranted in future clinical trials.^40,41^ We recruited across 4 sites, but the geographic diversity of the sample remains limited, which may limit generalizability. We were unable to measure group cohesion in the virtual groups, which may be an important change mechanism of PLF and should be studied in future trials. Pandemic-related disruptions, including changes in delivery modality, were not formally modeled and may have influenced results.

## Conclusions

In this RCT of 207 veterans, assignment to PLF did not reduce time to first instance of suicide behavior within an ITT framework but was associated with longer time to first actual suicide attempt. Although the finding for actual suicide attempt was not based on a prespecified primary outcome, it suggests that PLF may influence the severity or trajectory of suicide behavior. PLF was also associated with improvements in suicide-related coping and select secondary outcomes. While PLF was studied primarily in urban areas, its transition to virtual delivery during the pandemic allows extension to less populated and rural communities. Future PLF studies can target a wider geographic area and other veteran cohorts with elevated risk for suicide, including recently discharged service members and veterans not engaged in VA care.

## Notes

Supplement 1.
Trial Protocol

Supplement 2. **eMethods.** Project Life Force (PLF) Session Content **eTable 1.** Exploratory Outcomes **eResults 1.** Primary Outcome With a Modified Intent-to-Treat Sample **eResults 2.** Secondary and Exploratory Outcomes With a Modified Intent-to-Treat Sample **eTable 2.** Secondary Outcomes With a Modified Intent-to-Treat Sample **eTable 3.** Exploratory Outcomes With a Modified Intent-to-Treat Sample

Supplement 3.
Data Sharing Statement
