# Supplementary material for: Group-Based Suicide Safety Planning and Skills Training for Veterans With High Suicide Risk: A Randomized Clinical Trial
Source: JAMA Netw Open. 2026 May 29;9(5):e2615029. doi: 10.1001/jamanetworkopen.2026.15029 (PMC13221691; doi:10.1001/jamanetworkopen.2026.15029)
Supplement: Supplement 1. — Trial Protocol [file jamanetwopen-e2615029-s001.pdf]

B. PROTOCOL SUMMARY

1. Provide a brief (200-250 word) summary of background information for physician/scientists:

At present, we are unaware of any empirically supported, manualized outpatient group treatments for suicidal individuals other than a published account of a 1-session inpatient safety planning group and description of an outpatient process oriented group of variable session length and duration. Dialectical Behavior Therapy (DBT) offers a group component in its comprehensive approach, but discussion of suicidal urges, thoughts and behaviors is discouraged in the group setting. The paucity of published literature on group approaches for suicidal individuals is surprising given the numerous advantages of group treatment that include: 1) diminishing social isolation and increasing social support, a protective factor against suicide; 2) it's cost effectiveness and maximizing staff time; 3) the peer movement among those who have experienced suicidal crises is strong and growing; and 4) Veterans and military service members are used to working with a team approach to problems.

PLF is one of the first outpatient manualized suicide prevention group intervention to date. The proposed study has direct VA and military relevance as psychotherapy groups are a standard treatment modality in both settings.

2. State the purpose of the study:

The study proposes to conduct a randomized clinical trial (RCT) of a group safety planning intervention, "PLF" versus individual safety planning in 180 suicidal Veterans. Compared with the comparison condition- individual safety planning, the current standard of care, designated as treatment as usual (TAU), the PLF group will show:

3. Background of Study Population - Clinical Information:
   a. Describe the general population from which you plan to recruit subjects for this study (i.e., Demographics of potential subjects):

Male and female Veterans from the JJP VA Medical Center will be recruited, ranging in ages from 18-89. Participants should represent the spectrum of ethnicities and races, as well as socioeconomic status of the Greater New York area.

   b. Describe the medical condition in this population:

Veterans will have a history of suicidal ideation and suicide attempts (actual, aborted, or interrupted).

   c. How does this condition affect this population (symptoms)?

Models of suicidality stress the role of social factors in development and intensification of suicidal thoughts and behavior, including: feeling like a burden on family and friends feelings of isolation and not belonging (perceptions of diminished support from family/social network).

   d. Does this population have an understanding or knowledge of the medical condition being studied?

Yes, this population will have at least a general understanding of the condition of suicidality.

   e. What is the traditional treatment provided for this condition?

Behavioral psychotherapy, couple's therapy, and general safety planning with clinician.

   f. Explain how special needs that may arise with this population due to this medical condition are addressed (e.g. Additional care provided, supportive therapy, physiotherapy, behavioral therapy):

Special needs that may arise will be addressed through contacting resident providers and support staff equipped to treat Veterans with suicidal symptoms.

g. Describe how research subjects will be referred for appropriate care while they are enrolled in the study?

Research staff will inform subjects of appropriate care based on the subject's experience and on the research staff's own clinical judgment of the subject's wellbeing while enrolled. Examples include research staff informing subjects about enrolling in mental health services (i.e., psychiatrist, and psychosocial workers, Suicide Prevention Coordinators), providing information on the Rapid Access Clinic, and other resources such as substance abuse treatment and housing assistance that are available to all subjects.

h. Describe whether providers (all clinical staff) have experience in providing care to subjects:

The PI (Dr. Goodman) has extensive clinical and research experience with high risk suicidal Veterans, including the development of a DBT Clinical Program in VISN 3 since 2002, that now includes 7 sites across New York and New Jersey and is PI on two Department of Defense (DoD)-funded studies. The DoD research involves implementing a four-year RCT (with 18-month follow-up) of DBT for suicidal Veterans.

i. Does the James J. Peters VAMC have the appropriate medical or psychosocial resources required for any emergency needs for research subjects?

Yes.

4. Selection of subjects:

a. Indicate number of subjects to be enrolled at this site:
180

b. Indicate total number of subjects to be enrolled, if multisite study:
320

c. Are veterans being enrolled at this site?

**YES**	**X**	**NO**	

d. Are non-veterans being enrolled at this site?

**YES**		**NO**	**X**

If No, proceed to question 4.e.
Justify why non-veterans will be enrolled at this site:
Of all subjects to be enrolled at this site, what percentage will be non-veterans?

e. Describe the setting in which the research will be conducted (e.g., research office, clinic, lab, inpatient unit, outpatient unit):

Recruitment will take place via referrals from suicide prevention coordinators and Dr. Leo Sher, the attending of the inpatient unit; assessments will be done in the privacy of a research office. The group sessions will be held in a clinic room on hospital wings 3B or 6A.

f. What is the expected length of time a study subject will participate? (i.e., number of days, weeks, months, years):

12-14 months

5. Indicate the characteristics of study population (with the letter x in the appropriate column):

a. Gender:

	YES	NO
Male	X	
Female	X	

b. Age range: from **18** to **89** [max age is 89 years]

c. Racial and Ethnic/Minority Groups:

	YES	NO
Caucasian	X	
Alaskan Native	X	
American Indian	X	
Hispanic	X	
Black	X	
Asian/Pacific Islander	X	

Other: (Specify)

d. Justify any exclusion of specific gender, age, and racial or ethnic groups:
NA

6. State inclusion criteria for enrollment in study:

Inclusion Criteria include: 1. Age 18 years or older, 2. Discharge from inpatient unit for suicidal ideation or attempts, or placement on the high-risk suicide list maintained by suicide prevention coordinators, 3. Completion of a safety plan during the past 6 months prior to entry, 4. Concurrence from the patient's mental health provider for the Veteran to participate in the study and the provider is willing to work with the research team.

7. State exclusion criteria for enrollment in study:

Exclusion Criteria include: 1. Unable to provide informed consent or complete study requirements, 2. Unable to speak English, 3. Cognitive difficulties that impair consent Capacity, 4. Unable or unwilling to provide at least one verifiable contact for emergency or tracking purposes, 5. Unable to attend outpatient group treatment program or tolerate group therapy format, 6. Active alcohol or opiate dependence requiring medically supervised withdrawal, 7. Schizophrenia diagnosis, 8. Participation in another intervention RCT.

8. Will vulnerable subjects be enrolled in this study?

**YES**	**X**	**NO**	

	YES	NO
Individuals with lack of decision-making mental capacity		X
Economically/socially disadvantaged persons	X	
Educationally disadvantaged persons	x	
Pregnant women, for minimal risk only	X	

Fetuses Requires Medical Center Director authorization. See IRB SOP.
Children Requires Medical Center Director authorization. See IRB SOP.
Prisoners Requires CRADO authorization. See IRB SOP.

If Yes for any answer, provide a means by which vulnerability is determined: **Vulnerability will be determined through gathering basic demographic data of each participant. James J. Peters VA Medical has a population of veterans who are economically, socially, and educationally disadvantaged; thus, the PI anticipates recruiting from vulnerable populations.**

9. Are prospective subjects likely to feel vulnerable to coercion or undue influence to enter the study?

**YES**		**NO**	X

a. Are there any payments to subjects?

**YES**	X	**NO**	

If No, proceed to question 9.b.
How much are the payments? **$50-$75**
Provide a schedule (timing) of payments. **Baseline, post intervention, 3 months post completion of intervention, 6 months post completion of intervention**
In your opinion, will such payments have any influence on a person's decision to participate?

**YES**		**NO**	X

Why or why not? **These payments should not have any influence on a person's decision to participate because it is a relatively low amount meant to compensate the person for money spent on commuting, child care, taking off work, etc.**

b. What special safety precautions are utilized to minimize risk of coercion to protect the rights and welfare of these subjects [e.g., avoid financial incentives, consult with patient advocates or representatives]?

The PI will work closely with the providers and patient care representatives to ensure the rights and welfare of the subjects.

10. As PI, do you have access to sufficient population that will allow recruitment of the necessary number of subjects?

**YES**	X	**NO**	

11. Indicate how potential subjects will be recruited:
One hundred and eighty suicidal Veterans will be identified by primary clinician referral from the JJP VA psychiatric inpatient unit, or by the suicide prevention coordinator. All Veterans referred will be approached for consent or to participate in an information session about the study. This procedure will be followed in order of referral until the target number of cases have been consented and enrolled. Based on data from Dr. Goodman's completed DoD intervention study for suicidal Veterans, the estimated ethnic/racial breakdown of the study sample is 45% Hispanic, 48% White (non-Hispanic), 42% Black (non-Hispanic), 2% Asian/Pacific Islander, 2% American Indian and 6% multiple races.

12. Description of the Consent Process:
a. Describe the enrollment procedures for participation in the study:
Subjects who express interest will then be explained the study by one of the PIs and consent obtained. Prior to participation in the protocols, patients meet with research personnel at which time the nature of our program, the procedures involved, and alternatives to the study are discussed. The patient must understand and be able to articulate the risks and benefits of the research protocols and understand the alternatives. Patients who do not agree to participate in these protocols will

continue to receive clinical care in our facilities. Patients who agree to participate are reimbursed for their time and inconvenience at rates that do not represent inducement. After the protocol is described to the subject and the subject's questions are answered, the subject will be asked to summarize the procedures that he/she will undergo and to describe 2 risks involved in the study. If there is any doubt about the subject's capacity to give consent, an independent psychiatrist will assess the subject to determine whether they understand the study and can give informed consent.

c. Indicate when and where consent will be obtained:

Consent will be obtained upon meeting with the Veteran if s/he agrees to participate in the study. This meeting will take place in the privacy of a research office.

d. Who will conduct the consent interview?

Dr. Goodman, Dr. Kapil-Pair and/or Ms. Sullivan, Ms. Spears or Ms. Harris.

e. Will a legally authorized representative provide consent or permission for a subject, as appropriate?

**YES**		**NO**	**X**

e. What is the waiting period between informing the prospective subject about the study and obtaining consent?

As long as the Veteran needs to make decision.

Time devoted to consent discussion? **30-45 minutes**
Time allowed for a decision? **Indefinite**

f. How will you insure that the language used by those obtaining consent will not include exculpatory language which would waive or appear to waive any of the subject's legal rights?

The information being communicated to the participant during the consent process will not include exculpatory language through which the participant is made to waive or appear to waive any of the participant's legal rights. When the investigator verbally obtains consent, the participant will not be made to feel as if he or she has waived any of his or her rights as a human subject. The main purposes of the consenting process are for the investigator to inform the participant in detail about what will actually take place during the study, to allow the participant to ask any questions that he or she may have, and to explain to the participant that he or she is a volunteer for the study and may choose to decide against taking part at any point in time.

g. How will you insure that the information being communicated to the subject or the representative during the consent process will not include exculpatory language through which the subject or the legally authorized representative releases or appears to release the investigator, the sponsor, the institution, or its agents from liability for negligence?

We ensure that the information being communicated to the participant during the consent process will not include exculpatory language through which the investigator, the sponsor, the institution or its agents are released from liability by providing patients with contact information for the PIs and co-investigators should they feel that they have suffered an injury. Furthermore, patients are informed in the consent document that if they are eligible veterans, they are entitled to medical care and treatment if they experience any adverse effects resulting from the study. Participants are also provided with the contact information for the JJP VA IRB should they have questions regarding the research or their rights.

h. How will you know that the language is understandable to the prospective subject or the legally authorized representative?

The individuals communicating information to the participant during the consent process will provide that information in language understandable to the participant.

f. Indicate how you will determine whether the subjects (or their surrogates) understand the content of the information that is provided in the consent document:

After the protocol is described to the subject and the subject's questions are answered, the subject will be asked to summarize the procedures that s/he will undergo and to describe the risks involved in the study. A checklist will be used for this purpose by the person obtaining consent. If there is any doubt about the subject's capacity to give consent, an independent psychiatrist will assess the subject to determine whether s/he understands the study and can give informed consent.

g. How will you insure that the consent process will minimize the possibility of coercion or undue influence?

As mentioned above, upon hearing the description of the study, the participant will be required to describe the procedures to the person obtaining consent, summarize what is expected of him or her and list two risks involved. This will ensure that participants understand the implications of their decision to participate and are able to make a well-informed decision and communicate this decision to the person obtaining consent.

Consent will be obtained in a private setting by an investigator to minimize any power differential that the participant might feel. Participants unable to communicate with the investigator for any reason will not be included in the study. Only subjects having the capacity to give informed consent will be enrolled in the study. The recruitment process will not be coercive or unduly influencing, as participants enter the research study voluntarily and understand that they have the right to withdraw at any time. Subjects will be told that non-participation in the study will not affect any current treatments they are receiving at VA or elsewhere.

h. Summarize what actually will be done to the subjects during their participation in the study:

The subject will fill out the informed consent. They will then fill out a series of clinician interviews and self-report measures asking questions related to thoughts and feelings about suicide, general mental and physical health, and relationship with other. After these are completed half the group will be randomized to the PLF intervention arm in which Veterans will participate in the 10 weekly groups.

i. Provide a clear description of what is being done for research purposes and what is being done as part of "usual care":

For research purposes: Initial consult with research investigators and consenting, which will take approximately 30 – 45 minutes, completing assessments (1.5 hours) and answering interview-style questions pertaining to the research topic (30 minutes). Subject matter of the interview includes: communication quality with others, overall life satisfaction, frequency of suicidal ideation or urges, and reasons for attempting suicide.

"Usual care": Subjects will be able to continue all regular treatment outside of the study.

j. Provide a detailed **list of tests and procedures** that will be performed for research purposes (e.g., blood tests, urine tests, cultures, interviews, questionnaires, surgical procedures, cardiac catheterization, pulmonary function tests, X-rays, scans, etc) during the course of the study. **List must indicate tests and procedures only for subjects who sign a VA consent form, regardless of where tests and procedures take place**:

Test /Procedure:	Where:	Performed by Whom:	When:	# of times:
Columbia Suicide Severity Rating Scale (CSSRS)–current & since last visit version	Research office	Sarah Sullivan, A. Page Spears, Rachel Harris	0, 3, 6,12	4
Beck Depression Scale	Research office	Sarah Sullivan, A. Page Spears, Rachel Harris	0, 3, 6,12	4
Beck Hopelessness Scale	Research office	Sarah Sullivan, A. Page Spears, Rachel Harris	0, 3, 6,12	4
Self-report log based on the Modified Cornell Services Index (MCSI)	Research office	Sarah Sullivan, A. Page Spears, Rachel Harris	0, 3, 6,12	4
Brief Survey of Safety Plan Utilization (BSSPU)	Research office	Sarah Sullivan, A. Page Spears, Rachel Harris	0, 3, 6,12	4
Suicide-Related Coping Measure (SRCM)	Research office	Sarah Sullivan, A. Page Spears, Rachel Harris	0, 3, 6,12	4
Group Psychotherapy Process Measure* **PLF treatment arm only**	Research office	Sarah Sullivan, Marianne Goodman K. Nidhi Kapil-Pair	1,5,10 (weeks)	3
Buss-Perry Aggression Questionnaire	Research office	Sarah Sullivan, A. Page Spears, Rachel Harris	0, 3, 6,12	4
Reasons for Living	Research office	Sarah Sullivan, A. Page Spears, Rachel Harris	0, 3, 6,12	4
Attitudes Towards Suicide Scale	Research office	Sarah Sullivan, A. Page Spears, Rachel Harris	0, 3, 6,12	4
Insomnia Severity Index	Research office	Sarah Sullivan, A. Page Spears, Rachel Harris	0, 3, 6,12	4
Beck Lethality Scale	Research office	Sarah Sullivan, A. Page Spears, Rachel Harris	0, 3, 6,12	4
The Self-Injurious Thoughts and Behaviors Interview	Research office	Sarah Sullivan, A. Page Spears, Rachel Harris	0, 3, 6,12	4
Suicide Intent Scale	Research office	Sarah Sullivan, A. Page Spears, Rachel Harris	0, 3, 6,12	4
Mini-International Psychiatric interview (MINI)	Research office	Sarah Sullivan, A. Page Spears, Rachel Harris	0	1
PLF Intervention Sessions	Research office	Marianne Goodman K. Nidhi Kapil-Pair	Weekly	10

k. If laboratory tests are part of this protocol, will some or all study-related tests be performed outside this medical center?

**YES**		**NO**		**N/A**	**X**

l. Provide a statement that defines who will be financially responsible for the costs associated with participation in the study (e.g., examinations, procedures, drugs, devices, etc.) and a statement that defines what will be provided without cost to the subjects:

The patient will not incur any costs from participating in this study. The PLF intervention will be provided free of charge. In the event of an injury resulting from the research study the facilities of the Bronx VA will be made available to patients

e. <u>VHA Health Record</u> (If yes to (i) below or if clinical resources are to be used, a VHA health record must be created in CPRS [VHA Handbook 1200.05 #29.j]).

m. Will the research intervention possibly lead to physical or psychological adverse events?

**YES**	**X**	**NO**	

(ii) Do you have a process in place to include a copy of the signed informed consent and HIPAA authorization in the medical record for the type research described above for each subject?

**YES**	**X**	**NO**	

If yes, Please describe: **A copy of each consent is scanned into subjects' charts and a hard copy is retained in study records**

14. Risks:

n. Identify study risk:

No Risk	
Minimal Risk	
Greater Than Minimal Risk	**X**

o. Describe the steps you could take to minimize risk to subjects, *without changing the science* of the study:

No subject will be taken off of medication treatment for the purpose of entering the research. We have selected our study tasks carefully to maximize the likelihood of obtaining an answer. We have an adequate number of qualified staff members and the necessary facilities to conduct the research. When appropriate, patients will be provided with medical or psychiatric referrals.

p. Could you use the data obtained from "usual care" so that tests are not repeated for research purposes (to minimize risk to subjects)?

**YES**		**NO**	**X**

q. Describe the steps you could take to minimize risk to subjects *by changing the science* of the study or study design:

We could offer this treatment in an inpatient setting where there is 24 hour supervision of the Veteran, however, this would limit availability of subjects and change the focus of the intervention.

15. Clinical Trials: If this study is a clinical trial (Part I, question 8) answer a-e, as applicable:

Has a description of this clinical trial been recorded on http://www.clinicaltrials.gov?

**YES**		**NO**		**NA**	**X**

r. Provide a list of investigational drugs that will be administered:
NA

s. Is the investigational drug information record attached?

YES		NO		NA	X

If Yes, please attach VA Form 10-9012 (Investigational Drug Informational Record) if not a marketed drug. ***VA Form 10-9012 is required on all investigational agents where a drug manufacture's package insert in not available.***
If a marketed drug, provide name(s) of authorized prescriber(s):

c. Is an investigational drug used in research for new or different purposes compared to that which the FDA approved? (listed in drug insert)

YES		NO		NA	X

Is there an IND number for this drug? (IND # needed for new investigational drugs)

YES		NO	

IND number or other status (**submit validation from Sponsor or correspondence from FDA)**:
If there is no IND number, is there an FDA exemption? (If yes, please submit FDA correspondence indicating exemption)

YES		NO	

d. Will investigational devices be used: **NA**
Is there an IDE (Investigational Device Exemption) issued by the FDA?

YES		NO	

IDE number or other status (**submit validation or FDA correspondence**):
Is there an abbreviated IDE?

YES		NO	

If there is no IDE number, is there an FDA exemption (If yes, please submit correspondence from FDA.)?

YES		NO	

How would you determine the risk-benefit analysis for investigational devices? (Either Significant use device or Non-significant use device – Consult FDA for determinations)

e. Risks for Investigational Drugs/Device: **NA**
For investigational drugs, what are the possible risks associated with the study drug relative to possible interactions with medications likely to be used by subjects?
What will your approach be to such risks?
Will such subjects be excluded from the study?

Have you reviewed scientific literature or relevant FDA and Sponsor Advisories alerts or warnings relevant to this medication/device?

YES		NO	

If there was any new information, what were the possible risks to subjects based on this information?
If there was any new information, how were potential benefits for subjects affected by this information?

If new information is received from the FDA or Sponsor that may affect the conduct of this study, would you be willing to change the study design or study procedures?

YES		NO	

How will you inform subjects of any relevant information from the FDA or Sponsor Advisories?

What advice will you provide to subjects if there are questions on continuing participation in the study?

16. Research Safety:

a. Describe your Data Safety Monitoring Plan for subjects (3-4 sentences):

The study will include a DSMB composed of individuals unaffiliated with the study who have expertise in clinical trials, clinical research, and research in suicide. These include David Jobs, PhD from Catholic University who will serve as the DSMB chair. He has experience as the director of suicide prevention. Also serving on the board will be Alejandro Interian, PhD from the VA New Jersey HCS –Lyons Campus and Daniel Weiss, PhD the editor of the Journal of Traumatic Stress and a Professor of Medical Psychology at the University of California San Francisco. The DSMB will create reports yearly.

The study will also include a medical monitor Dr. Ken Cohen. The medical monitor will review all unanticipated problems involving risk to subjects or others, serious adverse events and all subject deaths associated with the protocol and provide an unbiased written report of the event. At a minimum, the medical monitor will comment on the outcomes of the event or problem and in case of a serious adverse event or death, will comment on the relationship to participation in the study. The medical monitor also will indicate whether he/she concurs with the details of the report provided by the principal investigator.

b. What information will you be collecting for AEs/SAEs and what is your plan for reporting these to the IRB?

Subjects will be monitored by a study psychiatrist for symptomatic assessment, status of clinical condition and for suicidal ideation. All clinical findings and concerns will be discussed with the PI and adverse events will be reported to the IRB according to institutional guidelines.

This study is likely to have adverse events related to suicide attempts. Safety monitoring for adverse events (AEs) will be conducted in real time by the Principal Investigators and the research coordinators. The PIs will supervise the research and respond directly to patient problems. In the case of AEs, all of them will be indicated on the source documentation for the specific adverse event report form. The PIs will determine the severity of the event, will assign attribution to the event, and will monitor the event until its resolution.

How will you monitor adverse events? **Adverse events will be monitored through regular contact with study participants, as well as contact with providers. Participants will be carefully monitored during the interview by the research staff who will be trained in noticing when the participant is uncomfortable or needs to take a break. In addition, research staff will follow-up via phone call with participants within a day of the interview to check in and assure the participant is not experiencing any delayed distress. Participants who demonstrated distress during the interview will be closely monitored through follow-up phone calls and the PI and RA will notify the primary provider of the participant's experience during the interview.**

c. Provide a plan for periodic review of safety of research subjects and data:

All risks and potential complications are discussed with the subjects during consent signing, and any new information regarding risks will be provided by phone immediately. Patients are monitored closely and should any unanticipated findings arise, the IRB will be notified. PI (MG) will be available to perform a suicide assessment at any point a rater or clinicians delivering PLF suspects that a subject is at risk for suicide as determined through clinical questioning or a research instrument assessing suicidal intent. The senior clinicians will ask questions regarding suicidal ideation and suicide plan and arrange for transfer to the psychiatric emergency room and/or inpatient stabilization at the JJPVA should imminent suicidal behavior manifest itself. All suicide attempts and deaths will be reported to the JJPVA and the DSMB assembled for this study.

d. Describe how you will determine whether the study should be continued in light of emerging information:

The PI will work in conjunction with the IRB to evaluate any emerging information and determine if and how the study should be continued.

e. Is there a Data Monitoring Committee (DMC) or a Data Safety Monitoring Board (DSMB)?

**DMC**		**DSMB**	**x**	**NO**	

If no, proceed to question 17.

Describe your DMC or DSMB, including frequency of reporting: **The DSMB will communicate with yearly written reports to the IRB.**

Describe how the DMC or DSMB will communicate with the IRB: **The DSMB will communicate with yearly written reports to the IRB. The DSMB has contact information such as e-mail for the IRB and knows how to reach out to them.**

17. Data Analysis and Sample Size:

a. Describe the analysis that will be performed on the data:

All data will be de-identified before it is entered into a redcap database at the JJPVA and Philadelphia VA over the intranet. All principal analyses will be conducted based on the intention-to-treat principle. Linear mixed models will be used to examine changes in primary and secondary outcomes for Veterans over time and to examine whether the effects of PLF and TAU on these outcomes vary with time adjusted for the time-dependent treatment, covariates of interest and imbalanced baseline prognostic factors, using data reduction methods as appropriate. *For the PLF subjects, individual's random effect will be nested within the group they are assigned to, while for TAU subjects, they will be nested within the clinician effect*. All data will be entered into an SPSS database at the JJPVA constructed by the data manager. Tenets of our data system include double data entry, range checks and exclusion of identifiers that can be directly traced to individuals. Preliminary analyses will include computing descriptive statistics and inspecting features and patterns of data to determine whether data transformations are needed. Quantitative measures will be graphed and their distribution inspected for any outlier value, which will be winsorized. All principal analyses will be conducted based on the intent-to-treat principle, that is, all Veterans with data will be included, regardless of the actual treatment received. For subjects who drop out during the study, their data up to that point will be used, but not carried forward to subsequent time points.

<u>Analyses to Determine Generalizability of Results</u>: We will compare the baseline characteristics (e.g., age, sex, education) of the study population to those that declined participation, and will evaluate reasons for refusal that will be tracked. A sensitivity analysis using the propensity score methodology would be applied if those who refuse participation were significantly different on important measures from the participants. Missing data and loss to follow-up are usually concerns in longitudinal studies presenting problems in interpretation. The validity of the resulting inferences depends critically on the underlying mechanism that generates the missing observation- MAR, MCAR or MNAR.44 Both survival analysis and mixed-effect models, the proposed statistical methodology, can handle data that is missing at random or missing completely at random; that is, if the mechanism for missing data does not depend on the value of the response variable, given the information in the predictor variables already in the model (treatment condition and baseline scores), then no further adjustment is necessary. However, when missing data are related to outcomes of interest even after conditioning on the known data (i.e. treatment condition), such as when in some patients side-effects of the active treatment affect the suicidal behavior risk and also cause drop-out, parameter estimates and resulting tests of hypotheses will be biased, and this can affect the generalizability as well as the reproducibility of the results. To address this problem, we will look for patterns of missingness. For example, data will be stratified according to their missing pattern (e.g., early and late termination, and incomplete follow-up); and, assuming a monotonic missingness pattern applies, we will use Diggle methodology,45 allowing us to examine the effects of missing-data patterns on outcome measures. We will explore other patterns using the techniques referenced above.

b. Provide power analysis or describe how sample size was determined?

For the survival analysis comparing the PLF and TAU treatment conditions, we assumed a 20% dropout in both groups based on previous safety planning data to date using SAFE-VET, exponential hazard functions, and considered four scenarios of suicide behavior reduction of PLF over TAU. We assumed a base rate of 49% for SB in the TAU group based on published data from ED-SAFE. The sample sizes needed for 80% power were calculated using the "Proc Power" procedure from SAS software © 2002- 2012. In the absence of pilot data, we used a published formula for the Variance Inflation Factor from the weighted log-rank test, to adjust the required sample size for within-group, and within-clinician, dependence. We assumed m=5 subjects per group or clinician, and an ICC for suicidal behavior within the group of r=0.01 based on literature. While power analyses were calculated for several reduction rates, we selected the 35% reduction rate as the most reasonable estimation for the reasons outlined below. The composite suicide behavior outcome has minimal data outside of ED-SAFE's reduction of 18% in non-veterans recruited from an emergency room. Stanley and Brown's SAFEVET data on Veterans presenting to an ED, reported a 45% reduction in suicide attempts (however the composite definition of suicidal behavior was not used). Moreover we anticipate PLF to have increased potency over SAFEVET due to the group setting and greater number of in-person sessions. Balancing the enhanced potency of the PLF intervention with the new composite definition of SB, the 35% reduction scenario is reasonable. Therefore, we expect the reduction to be above the minimum values indicated, and are confident that N=145 will provide ample power for this aim. Then, the research team included an additionally 35 to be consented who would likely not screen into the study or meet inclusion/exclusion criteria.

18. Community-based Participatory Research

Is this research based on Community-based participation?

YES		NO	X

If Yes, answer questions (18a-h). If No, proceed to Question 19.

a. Describe the community that will be involved in this research:

NA

b. What is the role of the community in this research?
NA

c. Does this research include a community organization that is engaged in research?

YES		NO	X

If Yes, does that organization have a Federal Wide Assurance (FWA)?

YES		NO	X

d. Describe any part of this community-based research that an outside IRB will review.
NA

e. Describe the risks to the community as a result of this research:
NA

f. Do you have a plan for disclosure of individual results to research participants?

YES		NO	X

If Yes, please describe:
NA

g. Describe who will have access to study data if multiple groups are involved:
NA

h. Is there an agreement for this research (e.g. CRADA, subcontract) or a data ownership agreement?

YES		NO	X

If Yes, please provide details: **NA**

19. Advertisements:

a. Are you submitting advertisements for IRB review? **Recruitment will not be through advertisements for this particular study.**

YES		NO	X

20. Privacy:

a. Explain the plan to protect Privacy and the Privacy Interests of subjects:
(1) Environment/Sharing of information (Time and place for study activities (e.g., potentially stigmatizing locations, such as a clearly identified pregnancy counseling center), nature of the information given, who receives and can use the information and whether information sought is of legitimate interest to the researcher):

Participants will meet with study staff in neutral conference rooms or offices that are private. The information sought will relate only to relevant demographic information (age, gender, race, employment, etc.), and the assessment questions will have to do with the research topic. If at any point a participant feels stigmatized or in distress, the study staff will immediately stop the interview and have the PI consult with the participant.

(2) Subject's expectation of privacy, comfort in the research setting, comfort with research procedures:

Once a Veteran consents to participate in the study, we shall first screen them to determine eligibility. We are only enrolling Veterans who regularly receive services from or work at the JJP VAMC, so the environmental experience will not differ from their usual routine.

b. Data Use:

Is data shared with external parties?

**YES**	**x**	**NO**	

If Yes, answer the following. If No, proceed to question 20.c.

Is there a Memo of Understanding with the external facility?

**PENDING**		**EXECUTED**	**x**	**NO**	

Is there a Data Use Agreement? (**NOTE: DUA needed for sharing/transferring data outside of VA. See the Privacy Officer regarding DUAs.)**

**PENDING**	**x**	**EXECUTED**		**NO**	

Indicate any other data agreements (specify):

**PENDING**		**EXECUTED**		**N/A**	**x**

c. Signature and Date:

Are subjects incompetent or do they lack decision making capacity?

**YES**		**NO**	**X**

If Yes, is there a signature line on the HIPAA authorization for the person legally authorized in writing by the individual or the individual's legal guardian?

**YES**		**NO**	

d. HIPAA Waiver:

Is a HIPAA waiver being requested?

**YES**	**X**	**NO**	

If No, proceed to 20e.

(a) Is there a written plan to destroy identifiers at the earliest opportunity consistent with conduct of the research?

**YES**	**x**	**NO**	

e. Specimens:

Are you collecting specimens (Blood, saliva, tissue, etc.) in this study?

**YES**		**NO**	**X**

Will you send specimens off site for analyses? (Material Transfer Agreement required)

**YES**		**NO**	**X**

If yes, please indicate how transporting specimens, who will transport and list name of institution, address and contact information for person(s) in charge of receiving, storing and processing of specimens at external site. Please also indicate how long specimens will be stored (for specimen storing), if all specimens will be used up or whether or not unused specimens will be destroyed, when they be destroyed, remaining specimens shipped back to PI at a later date, keep specimens stored indefinitely, whether or not specimens will be shared with other researchers (for specimen banking), for future analyses, etc. **NA**

If NOT sending specimens off site, please list procedure for storage, handling, by whom, destroying, length of time storing and whether or not specimens will be used for future research. **NA**

Does the study documentation state when specimens will be labeled with identifiable or de-identified information? **NA**

**YES**		**NO**	

Is a Material Transfer agreement attached? **NA**

**YES**		**NO**	

f. De-Identification of Data:
Does the protocol indicate whether or not data will be de-identified?

**YES**	**X**	**NO**	

If so, please indicate all that apply. If no, proceed to question 20.g.

De-identified information is provided to PI (who has access to IIHI) by his/her research team per a HIPAA authorization or waiver of authorization.	
De-identified information is provided by PI (who has access to IIHI) to his/her research team per a HIPAA authorization or waiver of authorization.	
De-identified information is to be sent to non-VA research team member, such as a statistician.	**X**
De-identified information will be disclosed to a non-VA party. If so, please identify:	

g. VA sensitive data
(a) Is VASI shared with parties external to JJP?

**YES**		**NO**	x

If Yes, is a Data Use Agreement (DUA) in place for this activity? (**NOTE: DUA needed for sharing/transferring data outside of VA. See the Privacy Officer regarding DUA's.)**

**YES**		**NO**		**N/A**	x

If N/A for DUA, give reason:

(b) Have all research staff who will transport, transmit, download, and/or store VA sensitive information (all formats and media, see VHA Handbook 1200.12, Definitions) outside of the VA protected environment obtained written approval from their immediate supervisor and JJP ISO (see the ISO for form "Authorization to Transport and Utilize VA Sensitive Information Outside Protected Environments"?

**YES**		**NO**	**X**

If yes, please name individuals who have received authorization:

(c) Collected or stored in hard copy outside of the James J Peters VAMC: **NA**

**YES**		**NO**	

If Yes, how will it be protected?:

(d) stored on non-VA computers or non-VA electronic media outside or inside the James J Peters VAMC: **NA**

**YES**		**NO**	

If YES TO ANY OF THE FOUR PRECEDING QUESTIONS, answer i-ii below:

i. Are VA sensitive data in hard copy shared with parties specified in the consent form?

**YES**		**NO**	

Are documents sent with a chain of custody (registered mail, FEDEX, UPS) or hand carried?

YES	x	NO	

If Yes, please specify:

ii. Is VA sensitive data sharing/transmission with parties specified in the consent form FIPS 140-2 compliant using encryption provided by VA (IRM)?

Transferred using data storage media (e.g., CD, USB flash "thumb" drive):

YES		NO	

If Yes, please specify type of media:

Transferred using electronic data transfer (e.g., email with PKI):

YES		NO	x

If Yes, please specify type of electronic data transfer:

h. Protection of Media Stored at Alternate Site:

Will VA Sensitive Information be removed from the VA Protected Environment?

YES		NO	x

Does the study team plan to store VA sensitive information outside the VA protected environment?

YES		NO	x

If Yes, by what method it will be protected?

i. Data Repositories:

Is any data collected for this study to be shared with any other research study?

YES		NO	x

Does this study use data that is collected under a separate research study?

YES		NO	x

If Yes, provide Protocol #: MIRB #: or non-VA entity.

NOTE: If you answered yes to (i) above, please submit the data repository application, data repository SOP, DUA(if applicable) and consent form from other study providing data (if applicable) to this one stating that data will be used for future research studies. See R&D Committee Procedures Manual, IRB SOP, and VHA Handbook 1200.12 regarding compliance with VA Research Data Repository requirements.

21. Information Security:

a. Software:

Will specially obtained software be used?

YES		NO	x

If Yes, please identify:

Source of the software

Will a license be required?

How will a license be funded?

What data will be stored in temporary files on computer hard drives?

b. Web applications: (used for such purposes as recruiting subjects, completing questionnaires or processing data)

YES	x	NO	

If Yes, please identify: **RedCap**

Web application's security features: **Security features of Redcap can be found following this link https://www.iths.org/wp-content/uploads/About-REDCap-Vanderbilt.pdf However, only De-Identified data will be analyzed using RedCap**

c. Incident Reporting:

Are procedures in place, in accordance with VA policy, for reporting incidents, i.e. theft or loss of data or storage media, unauthorized access of sensitive data or storage devices or non-compliance with security controls?

YES	x	NO	

If Yes, please describe the procedures, and briefly describe how they are conveyed to research staff. **The research staff should report to the PI's, who will then report to the proper authorities.**

DATA:
a. Data Flow:
Describe the data collection, data flow and/or data management process that will be used for the study: **Hard copy data is secured by the research coordinator and filed in locked cabinets in a locked office. Data that is entered electronically is stored on VA-secured server. No one outside of the research staff has access to these files. A password protected spreadsheet will link research ID number with patient identification, stored on the VA-secured server. Only the PI and research assistants will have access to this linkage file.**

b. Data Security Plan:
Will Electronic Data be stored?

YES	x	NO	

If Yes, please explain how it will be secured: **All electronic data is stored on VA-secured server. Any data entered on Recap will be De-Identified**

Will Paper records be stored?

YES	x	NO	

If Yes, please explain how it will be secured: **Hard copy data is secured in locked file cabinets in the research office.**

c. Data on a Hard Drive:
Will VA research data be stored on a computer other than the VA network?

YES		NO	x

If Yes, will it be encrypted?

YES		NO	

If Yes, will data that is stored outside the VA network be backed up regularly and securely stored on the VA network?

YES		NO	

d. Storage Location:
Identify share drive folder name where electronic data will be stored:
\\r04brxnas21.v03.med.va.gov\groups\MIRECC\Marianne Goodman\New PLF

Identify location (facility, room number) where hard copy data will be stored: **9B-50A/9B-70**

e. Data Transmission:
Will sensitive electronic information be transmitted? (Note: VA sensitive data or information may only be transmitted using VA-approved solutions such as FIPS 140-2 validated encryption)

YES		NO	x

If Yes, please provide the method of transmission:

f. Data Backup:
Will original electronic VA research data stored on a mobile device be backed up regularly and stored securely within VA's protected environment?

YES		NO		N/A	x

Will original electronic VA research data stored outside the VA protected environment be backed up regularly and stored securely within VA's protected environment?

YES		NO		N/A	x

g. Shipping Data:
Will hard copy, electronic research data, or any encrypted media be sent via delivery service with a chain of custody?

YES	x	NO		N/A	

If Yes, please describe chain of custody: **Any data that will be shared will be De-Identified and shared via Fedex.**
Is sensitive electronic research data that must be sent via common carrier (e.g., USPS, UPS, Fedex) encrypted with FIPS 140-2 validated encryption?

YES		NO		N/A	

h. Data Return (If no data is sent offsite, enter X here and proceed to i.):
What VA information will be returned to the VA? **None. The only data sent offsite will be De-Identified DVD recordings of sessions that will be broken once listened to for adherence.**
How will the information be returned to the VA? NA

i. Termination of Data Access:
Will access to research study data be removed for study personnel when they are no longer part of the research team?

YES	x	NO	

j. Per CRADO memo "Creation of Research Data Inventory to resolve OIG report 11-01823-294" of January 15, 2015, please submit a "Research Data Inventory Tool" to accompany this application. This tool may be found on the Research intranet page under Other Forms.
Is a form being submitted with this application?

YES	x	NO	

EQUIPMENT:
a. Will any portable, mobile or wireless devices be used for research purposes?

YES		NO	x

NOTE: a VA approved recorder will be used for adherence monitoring of group sessions.
If No, proceed to next section.
If Yes, are all these devices encrypted?

YES		NO	

How are these devices backed up?
Is the encryption FIPS 140-2 validated (required)?

YES		NO	

Has the investigator's immediate supervisor and the CIO authorized use of any such devices (contact the CIO for procedures)?

YES		NO	

b. Has all equipment owned by an affiliated institution, or purchased by such institution from grant funds, and used by the Investigator in this research project at JJP, been reported to the CIO (reporting to include: Type of equipment, Manufacturer, Model, Serial Number, PO Number, Vendor, Asset Value, Acquisition Date, Researcher's Service, and Location (Room Number)?

YES		NO	
