# Supplementary material for: Group-Based Suicide Safety Planning and Skills Training for Veterans With High Suicide Risk: A Randomized Clinical Trial
Source: JAMA Netw Open. 2026 May 29;9(5):e2615029. doi: 10.1001/jamanetworkopen.2026.15029 (PMC13221691; doi:10.1001/jamanetworkopen.2026.15029)
Supplement: Supplement 2. — eMethods. Project Life Force (PLF) Session Content eTable 1. Exploratory Outcomes eResults 1. Primary Outcome With a Modified Intent-to-Treat Sample eResults 2. Secondary and Exploratory Outcomes With a Modified Intent-to-Treat Sample eTable 2. Secondary Outcomes With a Modified Intent-to-Treat Sample eTable 3. Exploratory Outcomes With a Modified Intent-to-Treat Sample [file jamanetwopen-e2615029-s002.pdf]

Supplemental Online Content

Goodman M, Jager-Hyman S, Monahan M, et al. Group-based suicide safety planning and skills training for veterans with high suicide risk: a randomized clinical trial. *JAMA Netw Open*. 2026;9(5):e2615029. doi:10.1001/jamanetworkopen.2026.15029

eMethods. Project Life Force (PLF) Session Content
eTable 1. Exploratory Outcomes
eResults 1. Primary Outcome with a Modified Intent-to-Treat Sample
eResults 2. Secondary and Exploratory Outcomes with a Modified Intent-to-Treat Sample
eTable 2. Secondary Outcomes with a Modified Intent-to-Treat Sample
eTable 3. Exploratory Outcomes with a Modified Intent-to-Treat Sample

This supplemental material has been provided by the authors to give readers additional information about their work.

© 2026 Goodman M et al. JAMA Network Open.

eMethods. *Project Life Force (PLF) Session Content.*

Session Number	Session Content	Step of the Safety Plan
Session 1	Overview: Background, Introduction, VA Suicide Safety Plan, Group Rules, VA Crisis Line	Step 5
Session 2	Identification of Warning Signs	Step 1
Session 3	Developing Internal Coping Strategies & Learning to Better Manage Urges	Step 2
Session 4	Identifying People to Call for Help	Step 3
Session 5a	Sharing the Safety Plan with your Family,	Step 3 and/or 4
Session 5b*	Building Friendships	Step 3 and/or 4
Session 6a	Maximizing your Mental Health Treatment	Step 5
Session 6b	Making the Environment Safe	Step 6
Session 7	Increasing Access to the Safety Plan and Introduction to Suicide Recovery Mobile Applications	
Session 8	Improving your Physical Well-Being	
Session 9	Reasons for Living	List of Reasons for Living
Session 10	Recap, sharing of Safety Plans	
Add-on session*	Add on session: Dealing with the suicide death of a group member	

**Sessions noted with an asterisk are optional and are dependent upon group composition. Additionally, over the course of the study, there was increasing recognition and emphasis on the importance of lethal means safety, which prompted PLF therapists to deliver session 6b as a stand-alone session. Therefore, duration of treatment may have extended to twelve sessions (with the addition of sessions 5b and 6b) for select individuals.*

© 2026 Goodman M et al. JAMA Network Open.

eTable 1. *Exploratory Outcomes.*

	INQ-PB		INQ-TB	
Predictors	*Estimates (d)*	*p*	*Estimates (d)*	*p*
(Intercept)	18.84	**<0.001**	39.06	**<0.001**
Treatment [a]	0.20 (0.01)	0.898	-0.66 (-0.04)	0.718
Month 3	-2.08 (-0.18)	0.071	-3.08 (-0.19)	**0.049**
Month 6	-0.47 (-0.04)	0.696	-2.64 (-0.16)	0.103
Month 12	-2.99 (-0.26)	**0.010**	-3.90 (-0.24)	**0.014**
Site (JJPVAMC reference)				
CMJCVA	1.66 (0.15)	0.275	-0.00 (0)	1.000
CTVCS	0.88 (0.05)	0.724	-0.47 (-0.02)	0.865
Treatment x month 3	-1.54 (-0.09)	0.359	-1.33 (-0.06)	0.559
Treatment x month 6	-3.52 (-0.20)	**0.040**	-2.55 (-0.11)	0.271
Treatment x month 12	-1.83 (-0.11)	0.274	-2.61 (-0.11)	0.249
N	206 $_{id}$		206 $_{id}$	
Obs.	577		577	

Note. INQ = Interpersonal Needs Questionnaire-15, PB = Perceived Burdensomeness, TB = Thwarted belongingness, JJPVAMC = James J. Peters VA Medical Center, CMJCVA = Corporal Michael J. Crescenz VA Medical Center, CTVCS = Central Texas Veterans Health Care System.

[a]Treatment is operationalized as PLF + TAU (coded as 1) versus TAU (coded as 0).

© 2026 Goodman M et al. JAMA Network Open.

eResults 1. *Primary Outcome with a Modified Intent-to-Treat Sample*.

The modified intent-to-treat (ITT) sample consisted of seventy individuals who attended at least one session of Project Life Force (PLF) and the treatment-as-usual (TAU) sample (N = 106). Over the study period, 45 (25.57% of 176) individuals experienced a SB composite outcome including interrupted, aborted, actual SA or suicide death (29 in TAU, 16 in PLF). Of these, 40 experienced an actual attempt (28 in TAU and 12 in PLF). In the primary survival analysis with composite SB as the outcome, treatment condition was not a significant predictor of SB (HR = 0.80, 95% CI [0.43, 1.47], p = .47). Adjustments for site and lifetime SA were not significant (p-values > .05). In the post-hoc survival analysis, treatment condition was not a significant predictor (HR = 0.61, 95% CI [0.31, 1.12], p = .15). All model hazards were proportional (p-values > .05). The addition of gender as a covariate did not change these findings.

© 2026 Goodman M et al. JAMA Network Open.

eResults 2. *Secondary and Exploratory Outcomes with a Modified Intent-to-Treat Sample.*

The modified intent-to-treat (ITT) sample consisted of seventy individuals who attended at least one session of Project Life Force (PLF) and the treatment-as-usual (TAU) sample (N = 106). Treatment-by-time effects for depression, hopelessness and outpatient mental health utilization were non-significant (**Table S4-1**). Attitudes toward seeking professional psychological help significantly improved at the 3-month follow-up (p = .**033**) and positive expectations toward the future significantly improved at 1-year (p = .**015**) in PLF relative to TAU (**Table S4-1**). Overall suicide-related coping (SRC) significantly improved in PLF relative to TAU at 3-month follow-up (p = **.008**) (**Table S4-1**). External coping significantly improved at post-treatment (p = **.023**) and 3-month follow-up (p = **.041**), while internal coping significantly improved at 3-month follow-up (p = **.001**) for the PLF arm versus TAU. The Interpersonal Needs Questionnaire (INQ-15) subscales of thwarted belongingness and perceived burdensomeness did not significantly improve in PLF relative to TAU (**Table S4-2**).

© 2026 Goodman M et al. JAMA Network Open.

eTable 2. *Secondary Outcomes with a Modified Intent-to-Treat Sample.*

	BDI-II		BHS		BHS-P		SRCS		SRCS-E		SRCS-I		ATSPPH		Contacts	
Predictors	*Estimates*	*p*	*Estimates*	*p*	*Estimates*	*p*	*Estimates*	*p*	*Estimates*	*p*	*Estimates*	*p*	*Estimates*	*p*	*Estimates*	*p*
(Intercept)	30.80	**<0.001**	9.14	**<0.001**	5.54	**<0.001**	49.17	**<0.001**	20.22	**<0.001**	21.08	**<0.001**	21.73	**<0.001**	20.85	**<0.001**
Treatment [a]	1.18	0.619	1.36	0.198	-1.00	**0.043**	-1.10	0.554	-0.71	0.380	-0.01	0.989	1.02	0.246	2.00	0.527
Month 3	-7.26	**<0.001**	-1.81	**0.009**	0.41	0.215	1.65	0.218	0.68	0.253	0.40	0.456	-0.39	0.505	-0.68	0.745
Month 6	-5.29	**0.001**	-1.64	**0.022**	0.43	0.205	0.22	0.874	0.60	0.327	-0.35	0.528	-1.35	**0.026**	3.44	0.101
Month 12	-8.23	**<0.001**	-1.71	**0.015**	0.26	0.446	3.34	**0.014**	1.55	**0.011**	1.01	0.065	0.08	0.889		
Site (JJPVAMC reference)																
CMJCVA	-0.16	0.945	0.50	0.631	-0.24	0.617	1.10	0.536	0.66	0.382	0.51	0.425	1.18	0.173	-8.82	**0.002**
CTVCS	-2.65	0.494	-0.07	0.965	0.39	0.622	-1.53	0.598	-0.60	0.629	-0.47	0.655	-1.32	0.359	-6.53	0.154
Treatment x month 3	-0.52	0.824	-1.12	0.295	0.83	0.104	3.99	0.054	2.10	**0.023**	0.84	0.316	0.69	0.441	0.97	0.767
Treatment x month 6	-2.32	0.327	-1.22	0.264	0.73	0.161	5.64	**0.008**	1.93	**0.041**	2.72	**0.001**	1.95	**0.033**	3.41	0.299
Treatment x month 12.	-0.16	0.945	-1.56	0.148	1.26	**0.015**	3.73	0.075	1.61	0.085	0.96	0.256	0.82	0.366		
N	176 $_{id}$		176 $_{id}$		176 $_{id}$		176 $_{id}$		176 $_{id}$		176 $_{id}$		176 $_{id}$		169 $_{id}$	
Obs.	519		521		521		523		523		523		506		505	

Note. BDI-II = Beck Depression Inventory-II, BHS = Beck Hopelessness Scale, BHS-P = BHS positive expectation subscale; SRCS = Suicide-Related Coping Scale, E = External, I = Internal, ATSPPH = Attitudes Toward Seeking Professional Psychological Help, Contacts = Mental Health Treatment Contacts (see methods section for operationalization), JJPVAMC = James J. Peters VA Medical Center, CMJCVA = Corporal Michael J. Crescenz VA Medical Center, CTVCS = Central Texas Veterans Health Care System, Obs = number of observations. Significant values are **bolded**. *d* = Cohen's *d*.
[a]Treatment is operationalized as PLF + TAU (coded as 1) versus TAU (coded as 0).

© 2026 Goodman M et al. JAMA Network Open.

eTable 3. *Exploratory Outcomes with a Modified Intent-to-Treat Sample.*

	**INQ-PB**		**INQ-TB**	
Predictors	*Estimates*	*p*	*Estimates*	*p*
(Intercept)	18.60	**<0.001**	38.82	**<0.001**
Treatment [a]	1.11	0.522	-0.19	0.925
Month 3	-2.07	0.078	-3.06	0.051
Month 6	-0.47	0.701	-2.63	0.104
Month 12	-2.99	**0.012**	-3.87	**0.015**
Site (JJPVAMC reference)				
CMJCVA	2.48	0.146	0.55	0.769
CTVCS	0.82	0.769	0.36	0.909
Treatment x month 3	-0.99	0.587	-0.30	0.901
Treatment x month 6	-3.21	0.082	-1.08	0.661
Treatment x month 12.	-1.61	0.374	-2.75	0.257
N	176_{id}		176_{id}	
Obs.	515		515	

Note. INQ = Interpersonal Needs Questionnaire-15, PB = Perceived Burdensomeness, TB = Thwarted belongingness, JJPVAMC = James J. Peters VA Medical Center, CMJCVA = Corporal Michael J. Crescenz VA Medical Center, CTVCS = Central Texas Veterans Health Care System.
[a]Treatment is operationalized as PLF + TAU (coded as 1) versus TAU (coded as 0).

© 2026 Goodman M et al. JAMA Network Open.
