# Supplementary material for: Group-Based Suicide Safety Planning and Skills Training for Veterans With High Suicide Risk: A Randomized Clinical Trial
Source: JAMA Netw Open. 2026 May 29;9(5):e2615029. doi: 10.1001/jamanetworkopen.2026.15029 (PMC13221691; doi:10.1001/jamanetworkopen.2026.15029)
Supplement: Supplement 3. — Data Sharing Statement [file jamanetwopen-e2615029-s003.pdf]

Data Sharing Statement

Goodman. Group-Based Suicide Safety Planning and Skills Training for Veterans With High Suicide Risk. *JAMA Netw Open*. Published May 29, 2026.
doi:10.1001/jamanetworkopen.2026.15029

Data
Additional Information: Trial Registry: ClinicalTrials.gov Registry URL: https://clinicaltrials.gov/ Trial Registration Number: NCT03653637
Data available: Yes
Data types: Deidentified participant data
How to access data: marianne.goodman@va.gov
When available: With publication

Supporting Documents
Document types: None

Additional Information
Who can access the data: Researchers whose proposed use of the data has been approved.
Types of analyses: For pre-planned analyses, meta-analyses, and individual patient meta-analyses (if applicable)
Mechanisms of data availability: After approval of a proposal by the investigator team, with a signed data access agreement, and appropriate ethical approvals in place.
Any additional restrictions: None.
